# Control of the Intracellular Redox State by Glucose Participates in the Insulin Secretion Mechanism

**DOI:** 10.1371/journal.pone.0024507

**Published:** 2011-08-31

**Authors:** Eduardo Rebelato, Fernando Abdulkader, Rui Curi, Angelo Rafael Carpinelli

**Affiliations:** Department of Physiology and Biophysics, Institute of Biomedical Sciences, University of Sao Paulo, Sao Paulo, Sao Paulo, Brazil; Cardiff University, United Kingdom

## Abstract

**Background:**

Production of reactive oxygen species (ROS) due to chronic exposure to glucose has been associated with impaired beta cell function and diabetes. However, physiologically, beta cells are well equipped to deal with episodic glucose loads, to which they respond with a fine tuned glucose-stimulated insulin secretion (GSIS). In the present study, a systematic investigation in rat pancreatic islets about the changes in the redox environment induced by acute exposure to glucose was carried out.

**Methodology/Principal Findings:**

Short term incubations were performed in isolated rat pancreatic islets. Glucose dose- and time-dependently reduced the intracellular ROS content in pancreatic islets as assayed by fluorescence in a confocal microscope. This decrease was due to activation of pentose-phosphate pathway (PPP). Inhibition of PPP blunted the redox control as well as GSIS in a dose-dependent manner. The addition of low doses of ROS scavengers at high glucose concentration acutely improved beta cell function. The ROS scavenger N-acetyl-L-cysteine increased the intracellular calcium response to glucose that was associated with a small decrease in ROS content. Additionally, the presence of the hydrogen peroxide-specific scavenger catalase, in its membrane-permeable form, nearly doubled glucose metabolism. Interestingly, though an increase in GSIS was also observed, this did not match the effect on glucose metabolism.

**Conclusions:**

The control of ROS content via PPP activation by glucose importantly contributes to the mechanisms that couple the glucose stimulus to insulin secretion. Moreover, we identified intracellular hydrogen peroxide as an inhibitor of glucose metabolism intrinsic to rat pancreatic islets. These findings suggest that the intracellular adjustment of the redox environment by glucose plays an important role in the mechanism of GSIS.

## Introduction

Glucose is the main physiological stimulus for insulin secretion by pancreatic beta cells. Glucose oxidation increases ATP/ADP ratio, inducing the closure of the ATP-sensitive K^+^ channels (K_ATP_) and thus plasma membrane electrical activity [1], [2]. Membrane depolarization opens voltage-dependent calcium channels and promotes Ca^2+^ influx, triggering insulin granule exocytosis [3].

Although chronic exposure to high glucose is cytotoxic due to an excessive formation of reactive oxygen species (ROS) leading to oxidative stress [4], glucose is the main regulator of insulin secretion and it acutely enhances pancreatic beta cell function. Associated with this positive effect of glucose on beta cells, the cellular response to increasing glucose concentrations was reported to suppress, rather than stimulate, ROS production in rat pancreatic beta cells [5], [6]. In cultured purified rat beta cells, this glucose response correlates with activation of cell metabolism, as determined by the increase in the reduced state of the intracellular cofactors, NAD(P)H and FADH_2_/FMNH_2_ [6]. A recent study also proposed that ROS formation by cell metabolism can be overcome by the scavenging system that is supported by NAD(P)H generation [5]. Despite these findings, acute exposure to glucose has also been reported to increase ROS content [7], [8], [9].

ROS are constitutively produced and removed, driving a redox state that could act as signal for cell processes [10], [11]. It was recently shown that hydrogen peroxide (H_2_O_2_) stimulates insulin secretion in the presence of low glucose levels [5], [8]. On the other hand, at high glucose, this shift towards an oxidative state suppresses glucose stimulated-insulin secretion and its coupled mechanisms [12], [13], [14], [15]. The activity of enzymes involved in glucose metabolism, such as glyceraldehyde-3-phosphate-dehydrogenase (glycolytic pathway) and aconitase (Krebs cycle), have been reported to be inhibited by H_2_O_2_ [16], [17]. In this sense, the increase in the oxidative state by H_2_O_2_ addition was shown to impair glucose metabolism [15], decreasing the ATP/ADP ratio [18], which increases K_ATP_ channel activity and causes plasma membrane hyperpolarization [14], impairing intracellular calcium handling and insulin release [12], [15]. Therefore, the control of H_2_O_2_ content by glucose in pancreatic islets seems to be an important mechanism in cell function that still remains to be elucidated.

A possible pathway by which glucose could exert this control would be through activation of the pentose-phosphate pathway (PPP) as a source of NADPH, which is an important substrate for antioxidant defenses [4], [5]. Although there is conflicting evidence regarding the importance of PPP for NADPH production in islets [19], [20], [21], it has been recently shown that glucose-6-phosphate dehydrogenase (G6PD), the first enzyme in PPP, plays an important role in β-cell function and survival [4].

In the present study, we carried out a systematic investigation showing an effect of glucose on the redox balance of rat pancreatic islets. This effect is herein demonstrated to occur through the activation of the pentose-phosphate pathway, causing a reduction in intracellular ROS which are shown to have an inhibitory effect on glucose metabolism. Thus, the control of ROS content by glucose may also be considered a part of the process of glucose-stimulated insulin secretion.

## Results

### Glucose controls reactive oxygen species in rat pancreatic islets

**Figure 1:**
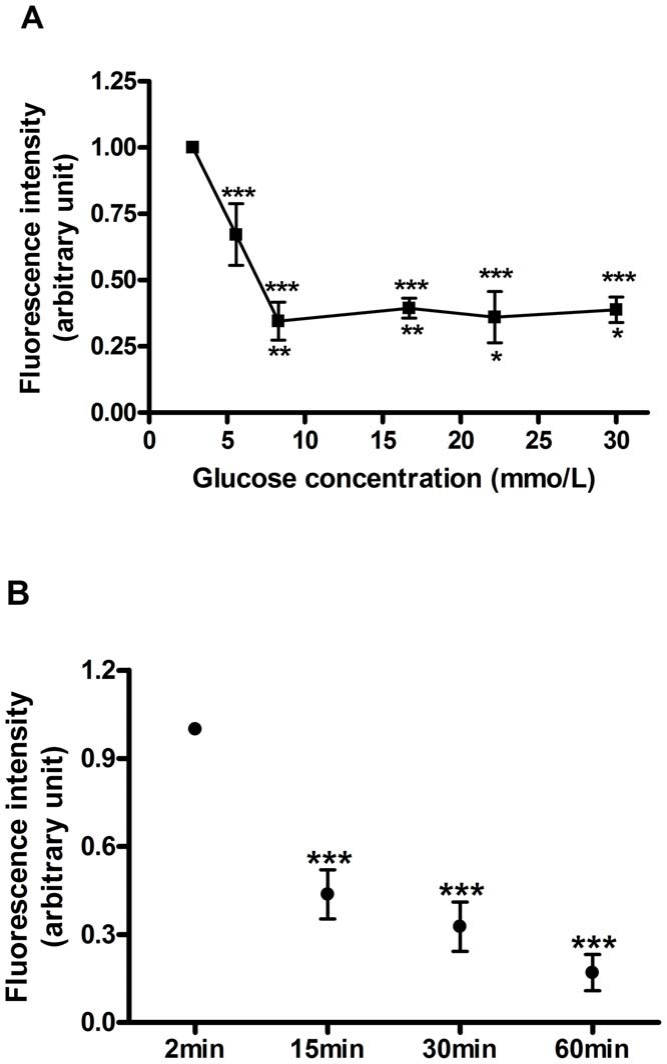
Effect of glucose on the intracellular ROS content. A) Islets were incubated for 30 minutes in the presence of different glucose concentrations: 2.8, 5.6, 8.3, 16.7, 22.2 and 30 mmol/L (2.8 mmol/L set as control condition). *p<0.05, **p<0.001 *vs* 5.6 mmol/L glucose, and ***p<0.0001 *vs.* 2.8 mmol/L glucose (n = 6). (B) Time-dependent effect of glucose: islets were incubated for different periods of time: 2, 15, 30 and 60 minutes, at 16.7 mmol/L glucose (2 min set as control condition). ***p<0.001 *vs.* 2 min (n = 3).

The concentration-dependent effect of glucose on intracellular ROS content was examined (Fig. 1A). Increasing concentrations of glucose (5.6, 8.3, 16.7, 22.2 and 30 mmol/L) suppressed the intracellular ROS content by 33±12, 65±7, 61±4, 64±10 and 61±5%, respectively, when compared with 2.8 mmol/L glucose (Fig. 1A). However, no statistical differences were observed between 8.3, 16.7, 22.2 and 30 mmol/L glucose. The suppressive effect of 16.7 mmol/L glucose on ROS content was also time-dependent (Fig. 1B). Significant decreases in ROS levels by 56±8, 67±8 and 83±6% were observed after 15, 30 and 60 minutes respectively, when compared to 2 minutes incubation (Fig. 1B).

**Figure 2:**
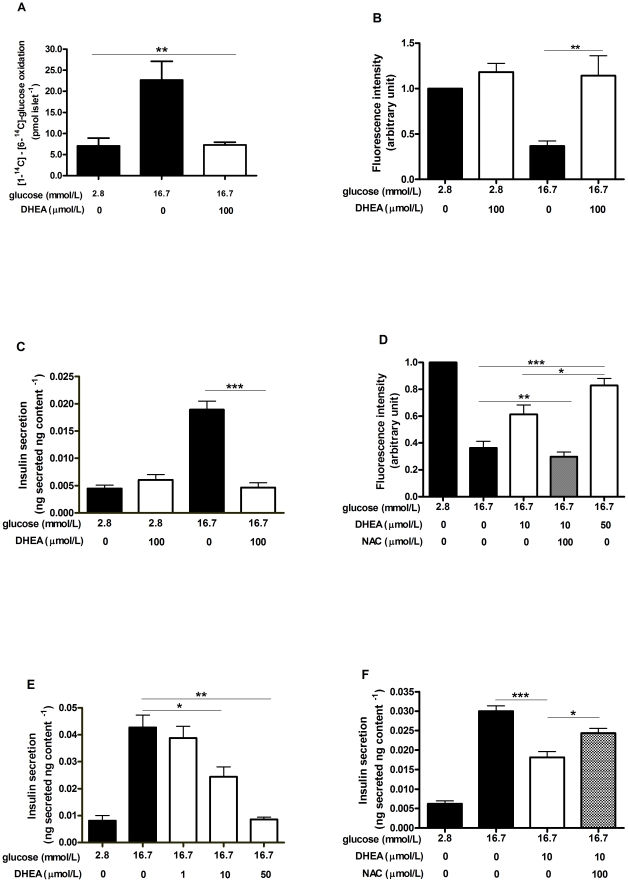
Involvement of the pentose-phosphate pathway (PPP) in the control of ROS levels and insulin secretion. Islets were incubated at 2.8 and 16.7 mmol/L glucose in the absence (black bars) or presence (white bars) of dehydroepiandrosterone (DHEA). A) Analysis of the PPP activity by the difference between [1-^14^C]-glucose and [6-^14^C]-glucose oxidation, after 60 minutes incubation (n = 4). B) Analysis of the intracellular ROS content in 30 minutes incubation (2.8 mmol/L set as control condition) (n = 6). C) Analysis of static insulin secretion at 60 minutes incubation (n = 8). D) Effect of lower DHEA concentrations on intracellular ROS content in the absence (white bars) or presence (dashed bar) of 100 µmol/L NAC in 30 minutes incubation (n = 5). E) Effect of lower DHEA concentrations on insulin secretion in 60 minutes incubation (n = 5). F) Effect of 100 µmol/L NAC (dashed bar) on the insulin secretion response to lower DHEA concentration in 60 minutes incubation (n = 7). *p<0.05, **p<0.01 and *** *p*<0.001.

A possible pathway through which glucose could exert this inhibitory effect on ROS content would be via its own metabolism in the pentose-phosphate pathway (PPP) with the consequent synthesis of NADPH, an important substrate for cellular antioxidant defenses. This way, dehydroepiandrosterone (DHEA), an inhibitor of glucose-6-phosphate dehydrogenase (G6PDH), the rate-limiting enzyme of the PPP [22], [23], was used to evaluate the involvement of this metabolic pathway in the effect of glucose on ROS control. The difference between the ^14^CO_2_ released from [1-^14^C]-glucose and [6-^14^C]-glucose indicates the absolute flux of glucose through the pentose-phosphate pathway [24], [25]. After 60 minutes of incubation, the difference between [1-^14^C] and [6-^14^C]-glucose oxidation was 7.09±1.8 at 2.8 mmol/L glucose and 22.68±4.4 pmol islet^−1^ h^−1^ at 16.7 mmol/L glucose (mean ± SEM). The addition of DHEA (100 µmol/L) to pancreatic islets in the presence of 16.7 mmol/L glucose markedly reduced (65±6%) the PPP activity to 7.28±0.6 pmol islet^−1^ h^−1^ (Fig. 2A). The absolute values of [1-^14^C] and [6-^14^C]-glucose oxidation are presented in Supplementary Table S1.

PPP inhibition abolished the intracellular control of ROS levels by 16.7 mmol/L glucose, raising ROS content (by 208±44%) to values similar to those observed at low glucose concentration (Fig. 2B). This effect was associated with a drastic impairment (by 75±4%) in the insulin secretion response to 16.7 mmol/L glucose (Fig. 2C). Lower doses of DHEA (10 and 50 µmol/L) promoted a dose-dependent impairment in ROS control by glucose, raising the ROS content at 16.7 mmol/L glucose by 83±27 and 125±47%, respectively (Fig. 2D). Associated with the inhibition of PPP activity (Fig. 2A) and with the failure in ROS control by glucose (Fig. 2B, D), the GSIS by pancreatic islets incubated in the presence of 16.7 mmol/L glucose was also suppressed in a dose-dependent manner by 43±9 and 80±2% respectively to the addition of 10 and 50 µmol/L DHEA (Fig. 2E). The possible involvement of ROS in mediating the effects of PPP inhibition on GSIS was assessed by using the antioxidant N-acetyl-L-cysteine (NAC −100 µmol/L), which prevented the increase in ROS content (Fig. 2D) concomitantly with a partial rescue of 52±10% (from 0.018±0.001 to 0.024±0.001 ng secreted ng content^−1^) on the inhibition of GSIS promoted by DHEA (Fig. 2F).

### Small increases in the antioxidant capacity improve pancreatic beta cell function

**Figure 3:**
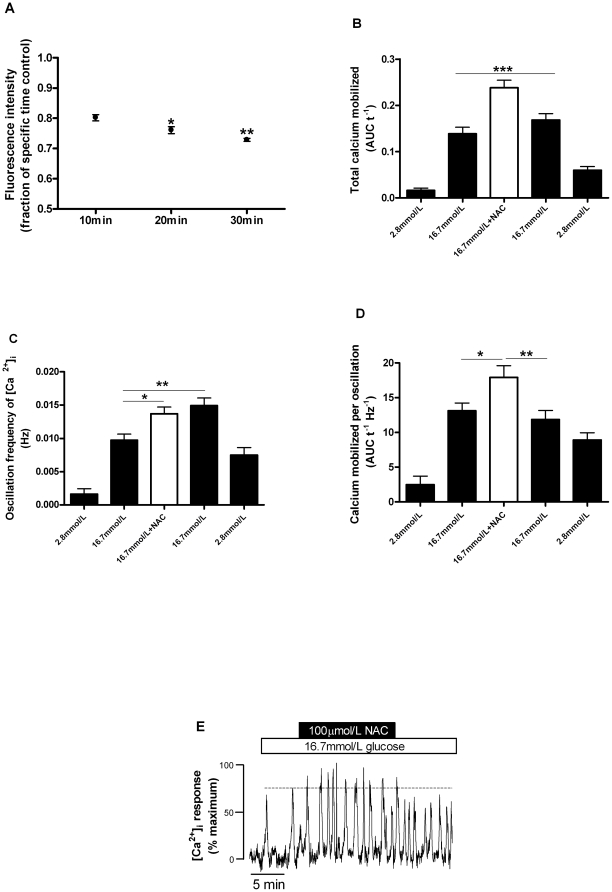
Effect of N-acetyl-L-cysteine on intracellular ROS content and calcium handling. A) Islets were incubated at 16.7 mmol/L glucose in the absence or presence of NAC (100 µmol/L) for different periods of time (10, 20, 30 minutes). The results are presented relative to controls without NAC for each different period (n = 3). *p<0.05 and **p<0.01 *vs.* 10 minutes period. Changes in intracellular calcium were observed in islets perifused with 2.8, 16.7 or 16.7 mmol/L glucose plus 100 µmol/L NAC (n = 33 cells from 14 islets from 5 animals): B) total intracellular calcium mobilized; C) frequency of calcium oscillations; D) calcium mobilized per oscillation; E) representative figure of NAC effect on [Ca^2+^]i, increasing the amplitude and frequency of calcium spikes. *p<0.05, **p<0.01 and ***p<0.001.

To evaluate the effect of ROS content on calcium handling, experiments using the readily membrane-permeable antioxidant NAC were performed, in which a significant decrease in ROS content of pancreatic islets was achieved (Fig. 3A). The effect of NAC was observed as a 20±1% reduction already in the first 10 minutes of incubation. Thereafter, the antioxidant effect of NAC slightly increased over the time-course, by 24±1 and 27±0.5% respectively to 20 and 30 minutes (Fig. 3A).

In association with the antioxidant effect observed already at 10 minutes of NAC addition, a positive effect on the intracellular calcium ([Ca^+2^]i) response to 16.7 mmol/L glucose was also observed. During islet perifusions, addition of 100 µmol/L NAC promoted an increase (by 72±13%) in the total calcium mobilized (Fig. 3B), together with an increase (by 41±8%) in oscillation frequency, which was sustained after NAC withdrawal (Fig. 3C). Also, the amount of calcium mobilized per calcium oscillation was enhanced (by 34±15%) during NAC treatment (Fig. 3D). Additionally, in 19 of the 33 cells studied the effect of NAC was also associated with an increase in the mean oscillation amplitude (28±8%), as Fig. 3E illustrates. Thus, these results show that a reduction in ROS content increases the amount of Ca^2+^ mobilized in islets.

**Figure 4:**
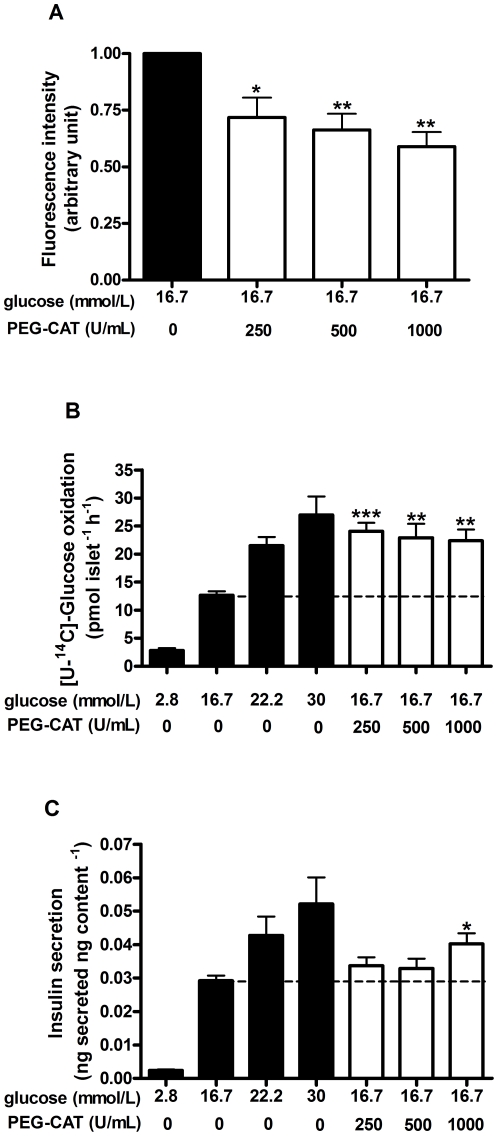
Effect of PEG-catalase (PEG-CAT) treatment on intracellular ROS content, glucose metabolism and glucose-induced insulin secretion. After pre-incubation (1 h) of pancreatic islets at 5.6 mmol/L glucose in the absence (black bars) or presence of PEG-CAT (250, 500, 1000 U/mL - white bars), the following experiments were performed where the islets were incubated with different glucose concentrations (2.8, 16.7, 22.2 or 30 mmol/L) without PEG-CAT. A) Analysis of the intracellular ROS content (16.7 mmol/L set as control condition) in 30 minutes incubation (n = 4). B) Analysis of [U-^14^C]-glucose oxidation in 60 minutes incubation (n = 5). C) Analysis of static insulin secretion in 60 minutes incubation (n = 6). *p<0.05, **p<0.01 and ***p<0.001 *vs*. 16.7 mmol/L glucose without PEG-CAT pre-incubation.

To further understand the role of the endogenous ROS in beta-cell function, changes in the redox state were also investigated by using a specific scavenger for hydrogen peroxide, catalase in its membrane-permeable form (PEG-CAT). Rat pancreatic islets pre-loaded with different PEG-CAT activities (250, 500 and 1000 U/mL) were assayed for ROS content, glucose metabolism and insulin secretion at 16.7 mmol/L glucose. The presence of PEG-CAT (250, 500 and 1000 U/mL) promoted an activity-dependent suppression in ROS content by 28±9, 34±7 and 41±6%, respectively (Fig. 4A). This was associated with an enhancement in glucose metabolism from 16.7 mmol/L glucose (12.7±0.7 pmol islet^−1^ h^−1^), to values similar to those observed at 22.2 (21.5±1.5 pmol islet^−1^ h^−1^) or 30 mmol/L (26.9±3.2 pmol islet^−1^ h^−1^) glucose. The effect of PEG-CAT on glucose oxidation over 16.7 mmol/L glucose was similar between the different activities of catalase (24.1±1.5, 22.9±2.5, 22.4±2.0 pmol islet^−1^ h^−1^, respectively to 250, 500 and 1000 U/mL – Fig. 4B). However, concerning insulin secretion, a positive effect of PEG-CAT over 16.7 mmol/L glucose (0.029±0.001 ng secreted ng content^−1^) could only be observed at 1000 U/mL (0.040±0.003 ng secreted ng content^−1^), reaching a value similar to the observed at 22.2 mmol/L glucose (0.043±0.005 ng secreted ng content^−1^) (Fig. 4C).

## Discussion

The importance of changes in the redox environment for glucose-stimulated insulin secretion was investigated in freshly isolated rat pancreatic islets. The effect of decreasing ROS levels by glucose and the involvement of the pentose-phosphate pathway in this process were also addressed.

A growing body of evidence points to the fact that acute changes in redox environment act as a signal for many cell processes [10], [11]. In the case of insulin secretion, this issue still remains unsolved. An increase in beta cell oxidative state impairs insulin secretion, as reported by several groups [4], [12], [13], [14], [15]. Although hydrogen peroxide at low glucose levels increases insulin secretion [5], [8], at high glucose the exposure of pancreatic islets to hydrogen peroxide reduces the ATP/ADP ratio [13], [18], hyperpolarizes the plasma membrane [14], impairs intracellular calcium handling [12], [15] and insulin secretion [12], [13], [15], [18].

Considering these negative effects of H_2_O_2_ on the beta cell response to glucose, a fine control of the intracellular ROS content might be required for GSIS. However, it was previously reported that high glucose stimuli were associated with an increase in ROS content [7], [9]. Despite that, analyzing ROS content in live cells of freshly isolated islets, we observed that glucose dose-dependently decreases ROS content in rat pancreatic islets (Fig. 1A) in a very fast manner (Fig. 1B). Similar results were observed in primary beta cells [6] and rat islets [5], where an increase in glucose concentration acutely resulted in a decrease in H_2_O_2_ content [5], [6], which correlated with a high cellular metabolic activity [6]. So, despite glucose can activate superoxide-producing NAD(P)H oxidase [26], the net effect of glucose during short periods of exposure amounts to an increase in antioxidant capacity [19], [27], thus reducing the overall ROS content in the process of glucose-stimulated insulin secretion.

This rapid suppressive effect of glucose on ROS (Fig. 1B) may be associated with a very fast increase in NADPH content and thus GSH/GSSG ratio, induced by high glucose in rat islets [19]. This suggests that the glucose-dependent effect on ROS involves metabolic changes that would increase the antioxidant capacity [6]. The pentose-phosphate pathway (PPP) is an important source of NADPH [23] and, although other sources of NADPH, such as the pyruvate malate shuttle, may be important in beta cells [20], a relevant role has been also attributed to PPP in the control of insulin secretion [21]. This is stressed by the recent finding that the impairment of insulin secretion in pancreatic islets due to glucotoxicity is mediated by oxidative stress derived from suppression of G6PDH expression [4], the rate-limiting enzyme of the PPP [22], [23]. In this study, it was observed that acute inhibition of G6PDH by DHEA reduces the carbon flux through the pentose-phosphate pathway (Fig. 2A). This PPP blockade abolished the effect of glucose-mediated ROS suppression (Fig. 2B) and also glucose-stimulated insulin secretion (Fig. 2C). Interestingly, this correlation between impairment in ROS control and decrease in insulin secretion (Supplementary Fig. S1) was also dose-dependent (Fig. 2D, E). The involvement of ROS in mediating GSIS inhibition can be evidenced by the presence of NAC, which restores the ROS content (Fig. 2D) and substantially prevents the GSIS impairment (Fig. 2F). These results indicate that PPP activity plays an important role during GSIS, partially by decreasing ROS content, but also possibly by direct stimulation of the exocytotic machinery by its metabolite [28].

Thus, despite their low antioxidant enzyme activities [29] and NAD(P)H oxidase activity [26], pancreatic beta cells are able to adequately deal with an increasing glucose supply [6], [30], ensuring ROS homeostasis. This redox control is achieved in response to increasing glucose concentrations, that raise the cellular antioxidant capacity by increasing the reduced glutathione (GSH) levels [19], the pentose-phosphate pathway activity (Fig. 2A) [19], [21], intracellular NAD(P)H content [28], [31] and glutathione peroxidase activity [27].

Despite that, intense depletion of H_2_O_2_ by loading cultured mouse islets and INS-1 cells with high concentrations of membrane permeable catalase (PEG-CAT) and NAC led to reduced GSIS. This was marginally reversed by KCl addition [8], which suggests an effect of H_2_O_2_ downstream of cell metabolism (i.e. activity of ion channels and/or exocytotic machinery). However, in our study the addition of a lower dose of NAC enhanced the [Ca^2+^]i response to high glucose (Fig. 3B, C, D), paralleled by a small decrease in the intracellular ROS content (Fig. 3A). Similarly, lower activities of PEG-CAT (250, 500, 1000 U/mL) associated with decreased ROS content (Fig. 4A) promoted a drastic increase in glucose metabolism (Fig. 4B), observed in all conditions tested. This shows that even the low intracellular levels of H_2_O_2_ observed at 16.7 mmol/L glucose can act as a negative regulator of glucose metabolism. Thus, in our study, treatment of rat islets with lower doses of antioxidants improved, rather than decreased, beta cell function in response to glucose. These results, together with those previously published by others, suggest that different levels of antioxidant supplementation lead to different responses in beta cells.

Despite the marked effect of all activities of PEG-CAT on glucose metabolism (Fig. 4B), insulin secretion was slightly changed only at the highest activity tested (Fig. 4C). Thus, glucose metabolism is more sensitive to redox changes (Fig. 4B) than other downstream events that contribute to insulin secretion (Fig. 4C). This uncoupling between metabolic and secretory effects of PEG-CAT at 16.7 mmol/L glucose may be due to the fact that at this glucose concentration the triggering mechanisms of insulin secretion are probably saturated. In effect, K_ATP_ conductance is abolished in glucose concentrations above 15 mmol/L [32].

In this sense, although no sharp differences among the PEG-CAT activities were observed by measuring the total cellular ROS content (Fig. 4A), a positive effect on GSIS was found at the highest PEG-CAT activity (Fig. 4C). This observation indicates that PEG-CAT at 1000 U/mL may affect local redox states, such as in plasma membrane regions containing NAD(P)H oxidase or in insulin granules, which are rich in the antioxidant enzyme glutaredoxin [28]. In effect, the knockdown of glutaredoxin has been demonstrated to impair insulin exocytosis [33]. Additionally, NAD(P)H oxidase is targeted to lipid rafts in the plasma membrane of endothelial cells [34] and, in beta cells, L-type calcium channels and the insulin exocytotic machinery are also localized in lipid rafts [35], [36]. This raises the possibility that local changes in the redox environment affect insulin exocytosis, which is a topic for future investigations.

In summary, these findings show that pancreatic islets adjust the intracellular ROS content according to the glucose supply and that this control plays a key role in the fine tuning of GSIS.

## Materials and Methods

### Ethics statement

This study was approved by the Animal Experimentation Committee of the Institute of Biomedical Sciences of the University of Sao Paulo, Sao Paulo, Brazil (permit number 107/2005) and followed the national guidelines for laboratory animal care.

### Animals and isolation of pancreatic islets

Female albino rats (150–200 g) were obtained from the Institute of Biomedical Sciences, University of Sao Paulo, Sao Paulo, Brazil. The animals were kept in groups of five at 23°C in a room with a light-dark cycle of 12∶12 h having free access to food and water. The pancreatic islets were isolated by collagenase digestion [37].

### Reagents

Type V collagenase, bovine albumin-fraction V, N-acetyl-L-cysteine (NAC), dehydroepiandrosterone (DHEA) and polyethyleneglycol-conjugated catalase (PEG-CAT) were purchased from Sigma Chemical Company (St. Louis, MO, USA). [1-^14^C]-Glucose, [6-^14^C]-glucose, [U-^14^C]-glucose and biodegradable scintillation liquid were obtained from Amersham (Little Chalfont, Bucks, UK). ^125^I-insulin was purchased from Perkin Elmer (MA, USA). Insulin antibody was a gift from Dr Leclercq-Meyer, Université Libre de Bruxelles, Belgium. 2′,7′-Dichlorodihydrofluorescein diacetate (H_2_DCF-DA) and Fluo-4-AM were purchased from Invitrogen (Eugene, OR, USA). D-Glucose and salts for buffer preparation were obtained from Labsynth (Diadema, SP, Brazil).

### Measurement of islet reactive oxygen species content

Groups of islets were incubated for 30 minutes (except in figure 1B when different times were tested) at 37°C in 500 µL of Krebs-Henseleit medium containing glucose and/or other testing substances as indicated in the figures and legends. H_2_DCF-DA at a final concentration of 5 µM was added and the islets, protected from light, were incubated for additional 20 minutes at room temperature. After this period, the islets were washed with Krebs-Henseleit buffer without glucose and analyzed by confocal microscopy using an excitation wavelength set at 488 nm and emission was collected through a 505–550 nm band-pass filter (LSM 510, Axiovert 100 M, Carl Zeiss; Germany) [38], [39], [40]. H_2_DCF-DA oxidation was quantified by the fluorescence emission intensity corrected for the islet area. The same confocal microscope parameters were used to analyze all samples in each independent experiment. For the measurements of ROS content, a control condition of each independent experiment was set as 1 arbitrary unit.

### Measurement of [^14^C]-glucose oxidation

Groups of islets were incubated in 2.4 mL of Krebs-Henseleit buffer containing albumin (0.2%), at 37°C in glass vials containing a filter paper and 400 µL of phenylethylamine, diluted 1∶1 v/v in methanol, in a separated compartment. The incubation buffer contained 36 µCi/mmol of [1-^14^C], [6-^14^C] or [U-^14^C]-glucose. This specific activity was maintained for all glucose concentrations tested. In the experiments to analyze [1-^14^C] and [6-^14^C]-glucose oxidation islets were incubated for 1 hour in the absence or presence of DHEA (100 µmol/L). In the experiments analyzing [U-^14^C]-glucose oxidation, islets were pre-incubated at 5.6 mmol/L glucose in the absence or presence of PEG-CAT (250, 500, 1000 U/mL) for 1 hour, followed by 1 hour incubation with different glucose concentrations. The incubations were stopped by the addition of 400 µL HCl (10 mol/L) and the vials were shaken for additional 90 minutes. The filter paper with phenylethylamine was transferred to a plastic tube with 1.8 mL of biodegradable scintillation liquid (Amersham Pharmacia, Uppsala Sweden) and the ^14^CO_2_ adsorbed was measured in a scintillation counter. Similar procedure was used in our previous studies [15].

### Static insulin secretion

Batches of 5 islets were incubated at 37°C in 500 µL of Krebs-Henseleit buffer containing albumin (0.2%) and different glucose concentrations. In the experiments to analyze the effect of DHEA islets were pre-incubated for 30 minutes at 5.6 mmol/L glucose, followed by 1 hour incubation in the absence or presence of different DHEA concentrations (1, 10, 50 and 100 µmol/L). In some experiments islets were also co-treated with 100 µmol/L NAC. In the experiments to analyze the effect of PEG-CAT on insulin secretion, islets were pre-incubated for 1 hour at 5.6 mmol/L glucose in the absence or presence of PEG-CAT (250, 500, 1000 U/mL), followed by 1 hour incubation with different glucose concentrations. After incubation, the medium was retrieved, frozen and later assayed for insulin by radioimmunoassay. After the secretion assay, the islets were disrupted in ethanol-water-HCl solution (52∶17∶1 v/v) and the intracellular insulin content was also measured. The insulin content was not different between the groups tested. The results of insulin secretion were calculated as the insulin secreted per total insulin content in the islets.

### Intracellular calcium measurements

Freshly isolated rat islets were loaded with fluo-4-AM (2.5 µmol/L) in RPMI-1640 medium for 3 hours at room temperature. Islets were allowed to adhere to poly-L-lysine pre-treated glass coverslips mounted inside a heated chamber (37°C) on the stage of an inverted confocal microscope (LSM510 Axiovert 100 M; Carl Zeiss, Jena, Germany). The preparation was then continuously perifused with Krebs-Henseleit buffer containing 2.8 mmol/L glucose (initial, 3 minutes), followed by 16.7 mmol/L glucose (29 minutes) and 2.8 mmol/L glucose in the last 5 minutes. During the 16.7 mmol/L perfusion, after the 4 initial minutes, NAC (100 µmol/L) was added for 15 minutes. Islets were excited at 488 nm, and emission was collected through a 505–550 nm band-pass filter [41]. Images were collected at 2 s intervals. Increases in [Ca^2+^]i are displayed as upward deflections. Individual β-cells were selected as regions of interest that responded to high glucose with a rise in [Ca^2+^]i (n = 33 cells from 14 islets from 5 animals). The [Ca^2+^]i responses were determined by normalization to the highest fluorescence peak after baseline subtraction using Origin 7.0 software (OriginLab, Northampton, MA). Quantification of the total calcium mobilized by the stimuli was performed by the analysis of the area under the curve normalized by the duration of each treatment (AUC t^−1^). The frequency of calcium oscillations in hertz (Hz) was analyzed by counting the number of upward deflections whose peaks were larger than 20% of the highest oscillation. The amount of calcium mobilized per oscillation, expressed by AUC t^−1^ Hz^−1^, was obtained dividing the AUC t^−1^ by the frequency.

### Statistical analysis

Results are presented as means ± SEM. Statistical analysis was performed by One-way ANOVA and Tukey's or Dunnett post-test as appropriate. Differences were considered significant for *p*<0.05.

## Supporting Information

Figure S1 **Pooled values of changes in ROS content (** **Fig. 2B and D** **) and insulin secretion (** **Fig. 2C, E and F** **) presented in parallel.** For ROS content (red circles) the values of 2.8 mmol/L glucose condition were set as 100%, while for insulin secretion (black squares) the values of 16.7 mmol/L glucose condition were set as 100%. Values are mean ± SE for 5–20 separate experiments.
(TIF)

Table S1 **Absolute values of [1-^14^C] and [6-^14^C]-glucose oxidation for the determination of the carbon flux through the pentose-phosphate pathway.**
(DOC)
