# Supplementary figures and images for: Control of the Intracellular Redox State by Glucose Participates in the Insulin Secretion Mechanism
Source: PLoS One. 2011 Aug 31;6(8):e24507. doi: 10.1371/journal.pone.0024507 (PMC3164208; doi:10.1371/journal.pone.0024507)

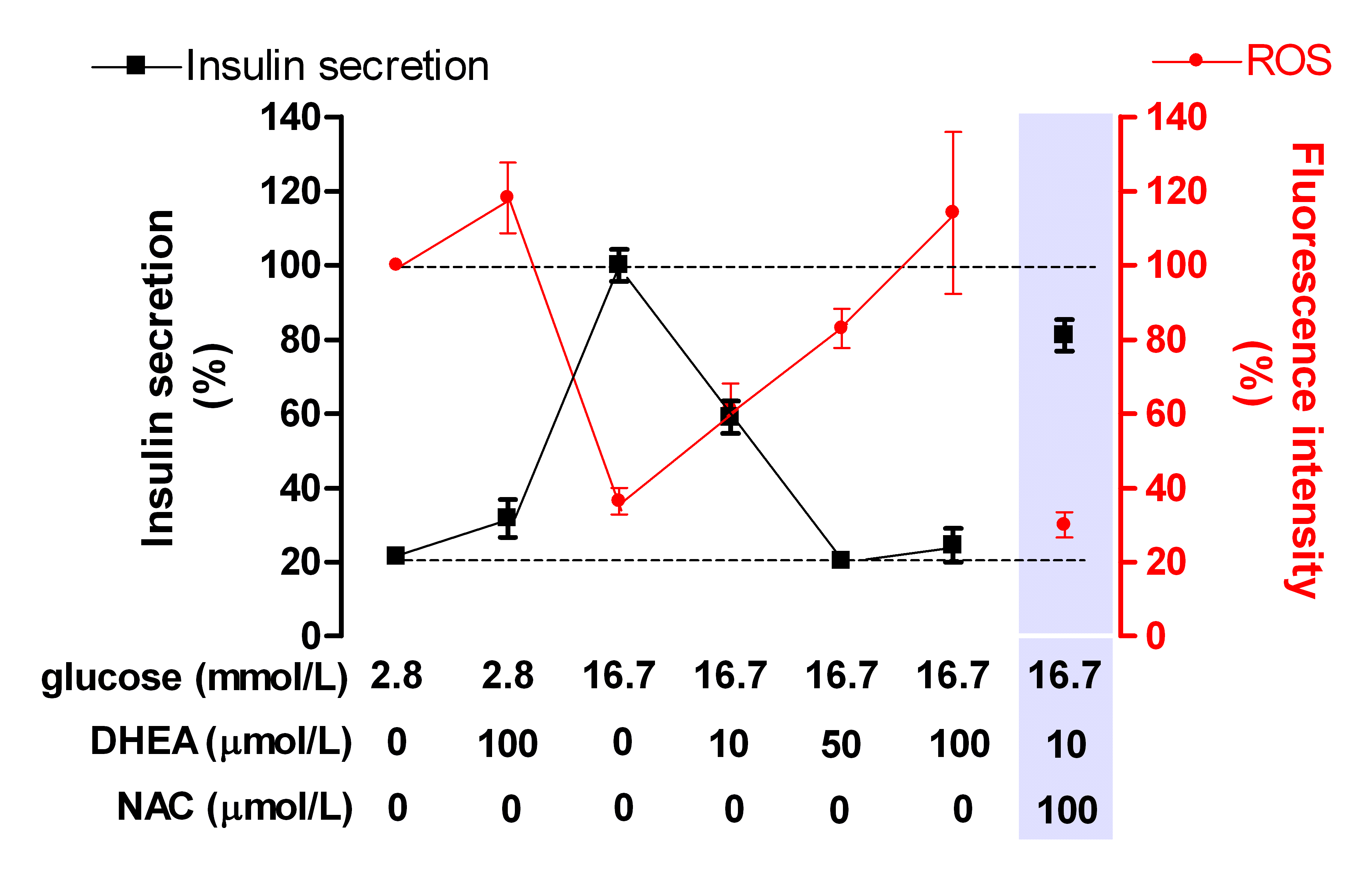

Supplement: Figure S1 — Pooled values of changes in ROS content ( Fig. 2B and D ) and insulin secretion ( Fig. 2C, E and F ) presented in parallel. For ROS content (red circles) the values of 2.8 mmol/L glucose condition were set as 100%, while for insulin secretion (black squares) the values of 16.7 mmol/L glucose condition were set as 100%. Values are mean ± SE for 5–20 separate experiments. (TIF) [file pone.0024507.s001.tif]
