# Supplementary material for: Control of the Intracellular Redox State by Glucose Participates in the Insulin Secretion Mechanism
Source: PLoS One. 2011 Aug 31;6(8):e24507. doi: 10.1371/journal.pone.0024507 (PMC3164208; doi:10.1371/journal.pone.0024507)
Supplement: Table S1 — Absolute values of [1-14C] and [6-14C]-glucose oxidation for the determination of the carbon flux through the pentose-phosphate pathway. (DOC) [file pone.0024507.s002.doc]

**Table S1** Absolute values of [1-^14^C] and [6-^14^C]-glucose oxidation for the determination of the carbon flux through the pentose-phosphate pathway.

|  | *2.8mmol/L* | | *16.7mmol/L* | | *16.7mmol/L*  *+ DHEA* | |  |
| --- | --- | --- | --- | --- | --- | --- | --- |
| [1-^14^C]-glucose oxidation  (pmol islet^-1^ h^-1^)  [6-^14^C]-glucose oxidation  (pmol islet^-1^ h^-1^) | | 10.6 ± 2.7  3.5 ± 1.0 | | 27.8 ± 4.9 *^a^*  5.3 ± 0.7 | | 11.9 ± 1.7 *^b^*  4.3 ± 1.0 | |

Values are mean ± SE for 4 separate experiments. See text for definition of abbreviations. *^a^p*<0.001 vs 2.8mmol/L and *^b^p*<0.001 due to DHEA effect.
